# Supplementary material for: Polyphenol Profiling of Chestnut Pericarp, Integument and Curing Water Extracts to Qualify These Food By-Products as a Source of Antioxidants
Source: Molecules. 2021 Apr 17;26(8):2335. doi: 10.3390/molecules26082335 (PMC8073822; doi:10.3390/molecules26082335)

Polyphenol profiling of chestnut pericarp, integument and curing water extracts to qualify these food by-products as a source of antioxidants

Supplementary Materials

Supplementary Materials

Figure 1 - pH profile of medium during the water curing with different treatments

A) tap water containing *L. pentosus*
B) tap water
C) recycled wastewater from a previous curing treatment with tap water

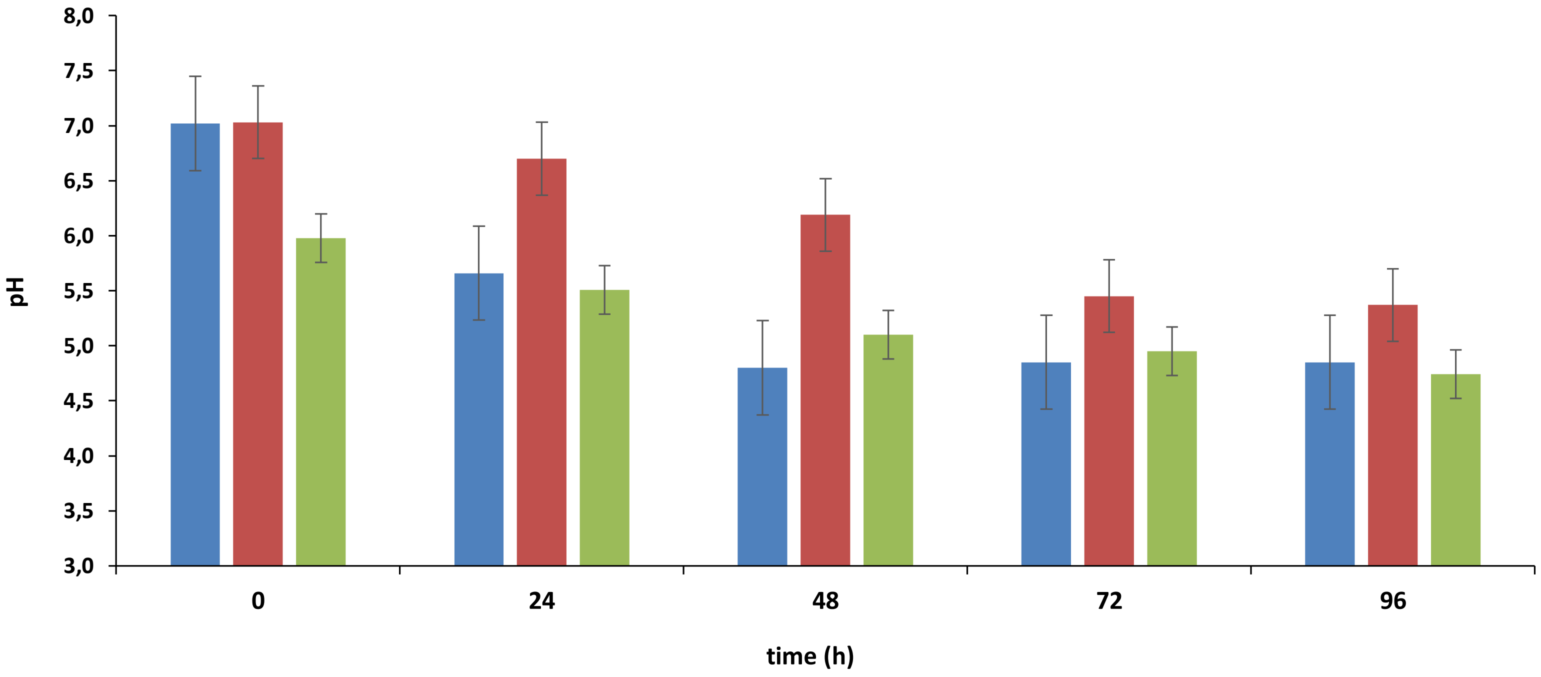

Supplementary Materials

Figure 2 - MS/MS spectrum at *m/z* 579 acquired in positive ion mode

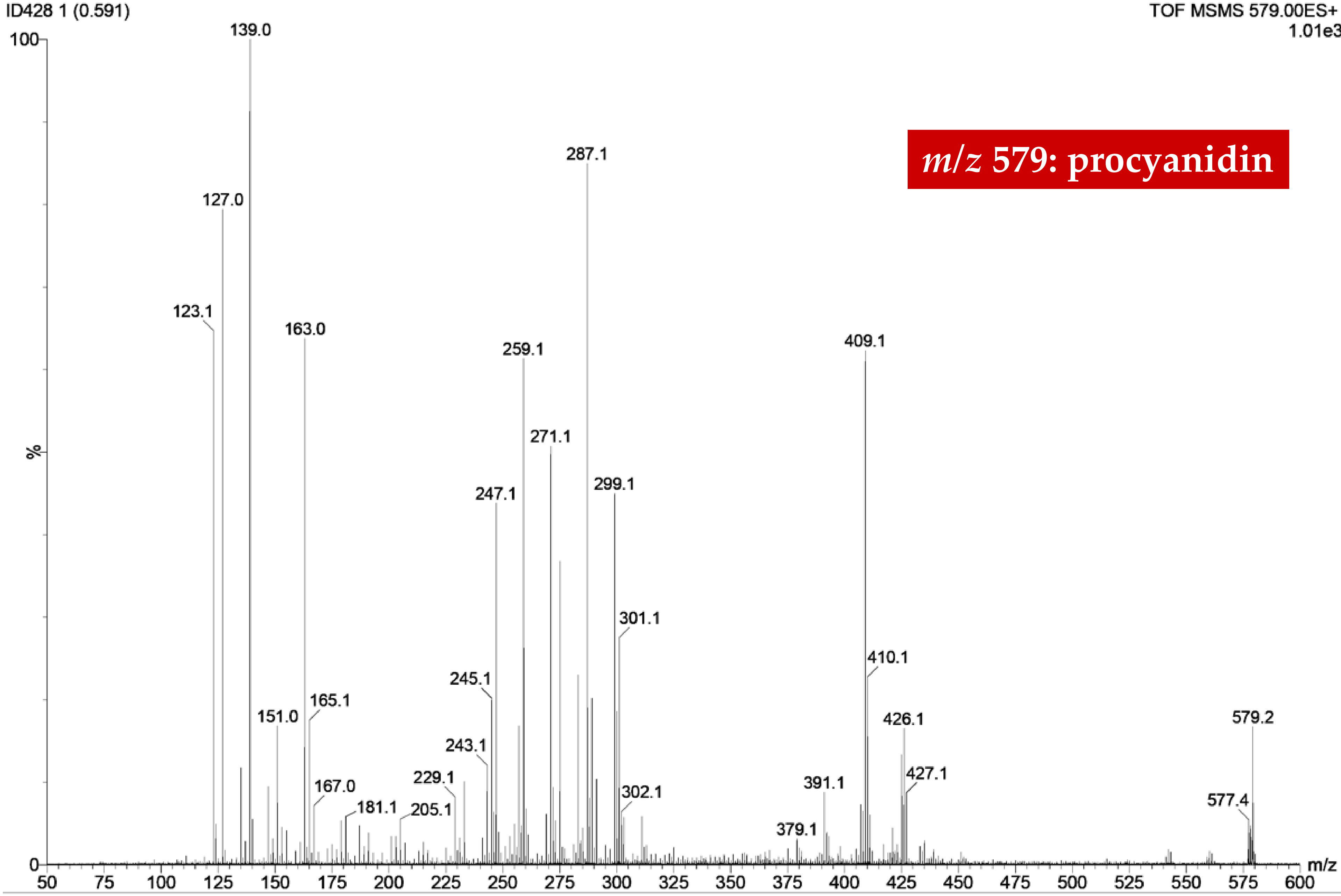

Supplementary Materials

Figure 3 - MS/MS spectrum at *m/z* 595 acquired in positive ion mode

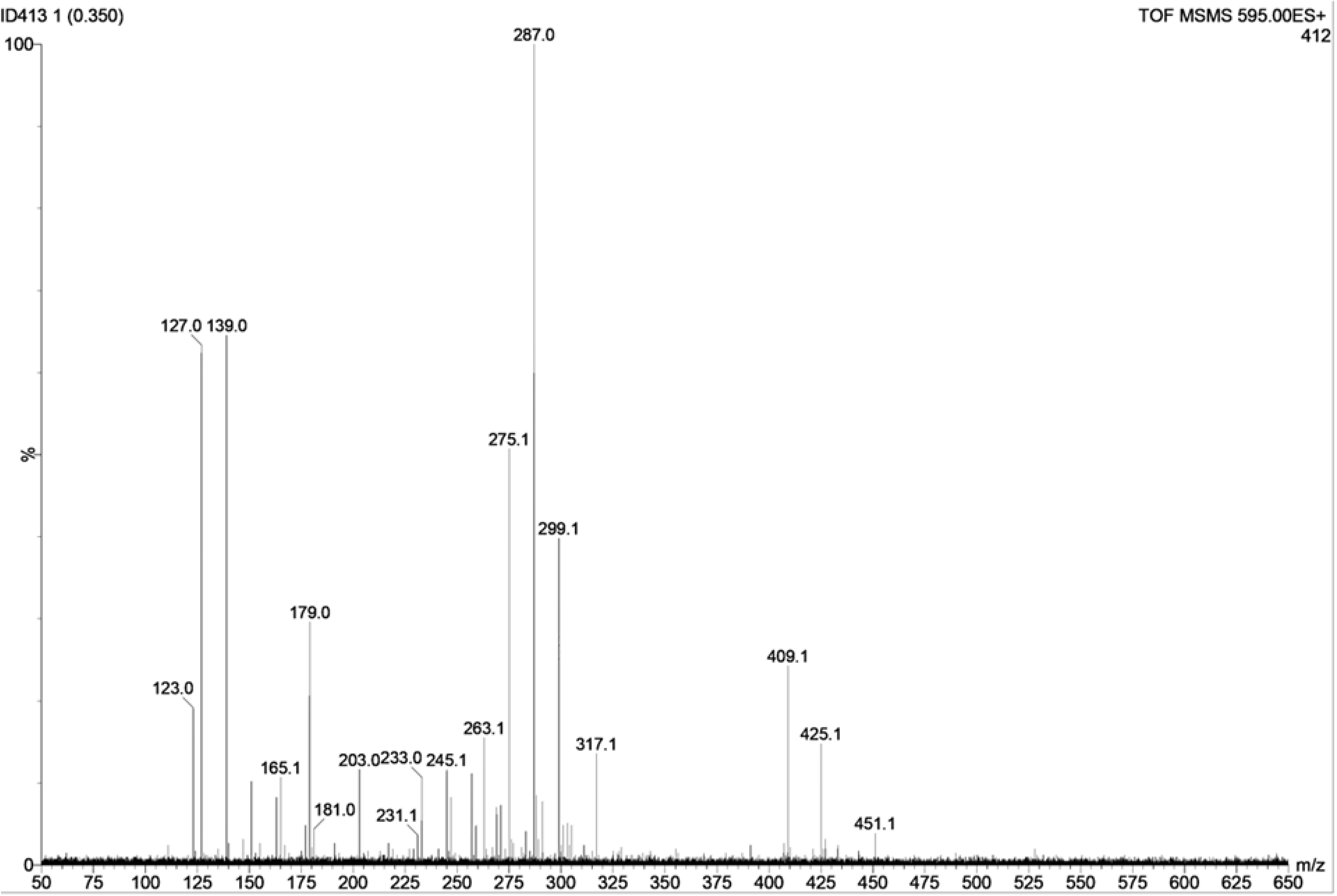

Supplementary Materials
Figure 4 - MS/MS spectrum at *m/z* 609 and 611 acquired in positive ion mode

m/z 609 and 611: dimeric (epi)gallocatechin A- and B-type

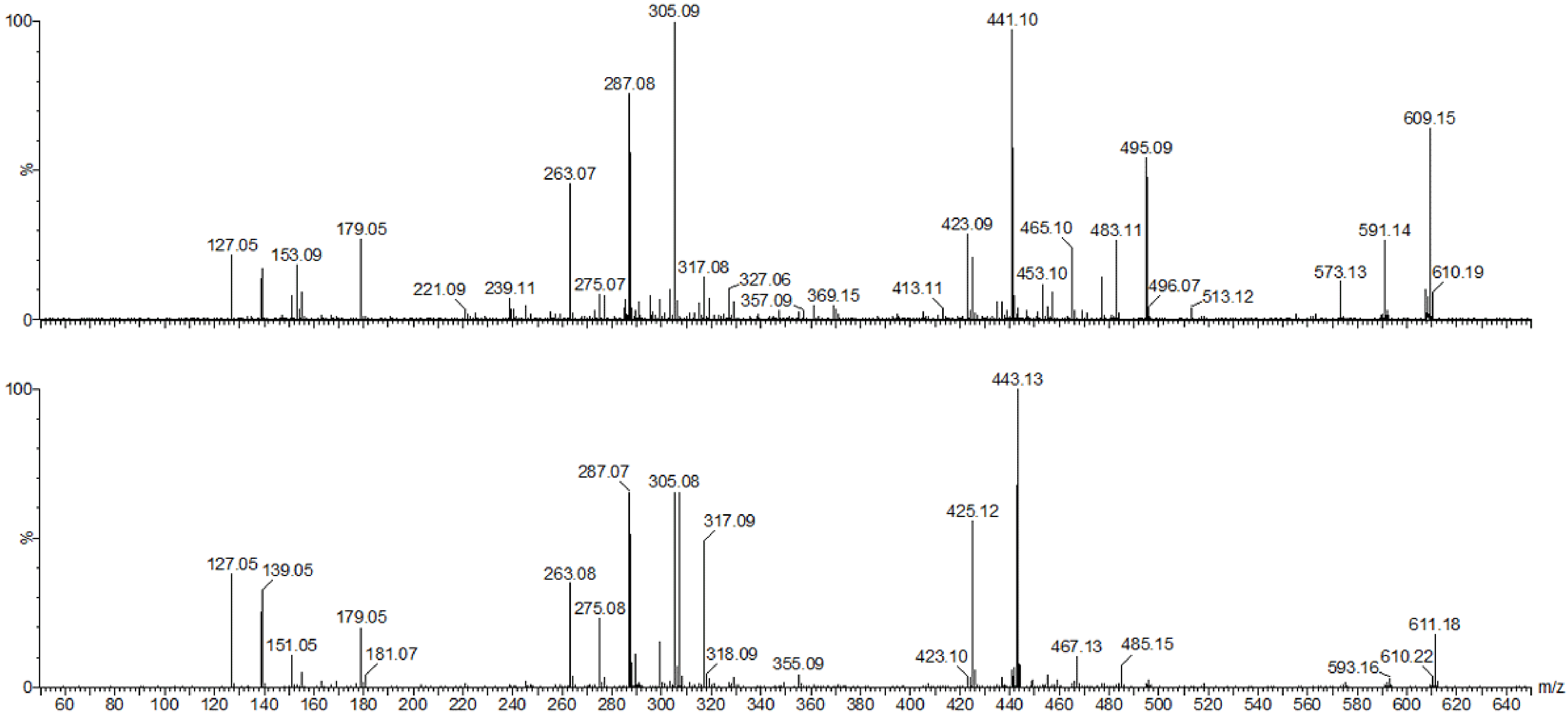

Supplementary Materials

Figure 5 - MS/MS spectrum at *m/z* 731 acquired in positive ion mode

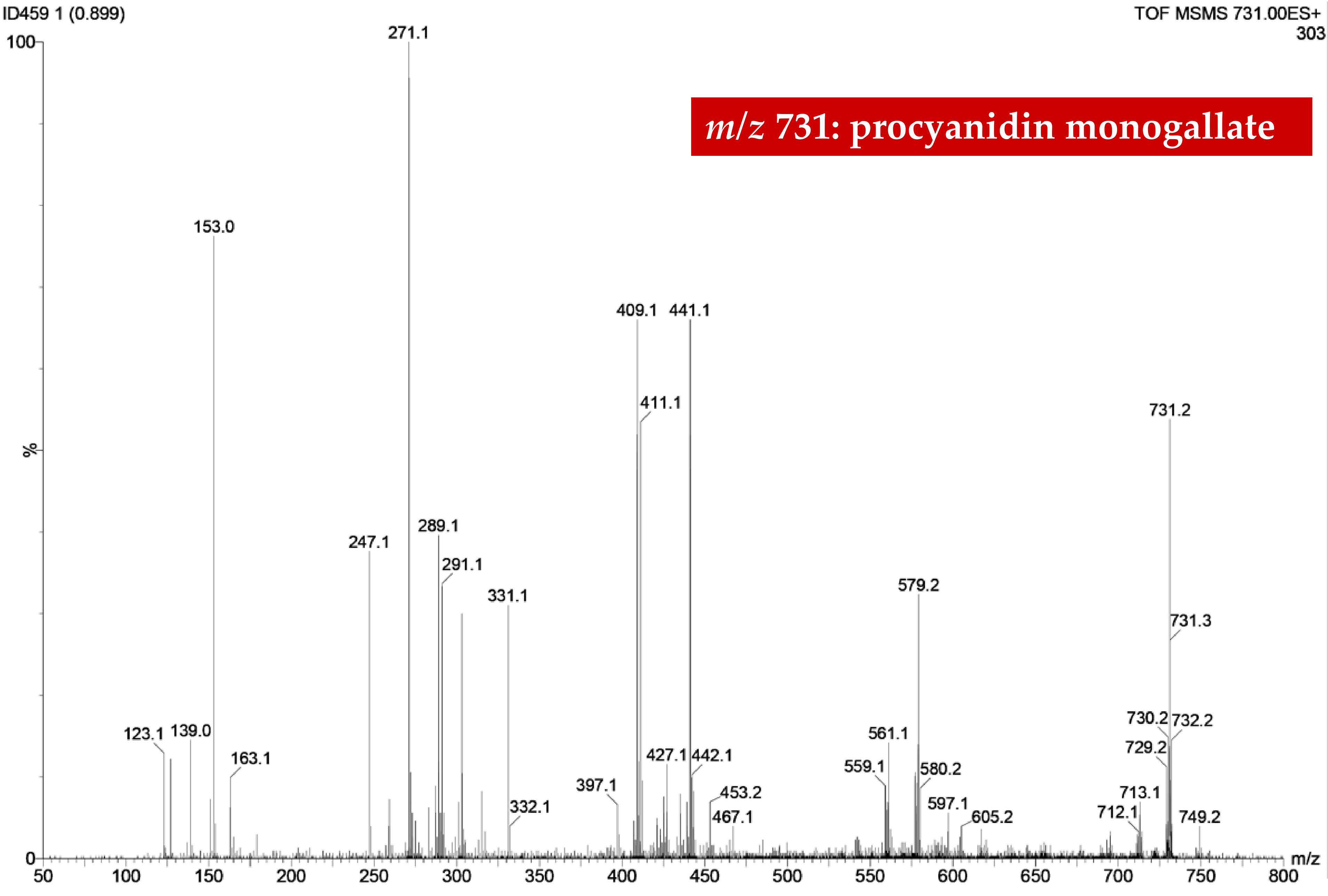

Supplementary Materials

Figure 6 - MS/MS spectrum at *m/z* 867 acquired in positive ion mode

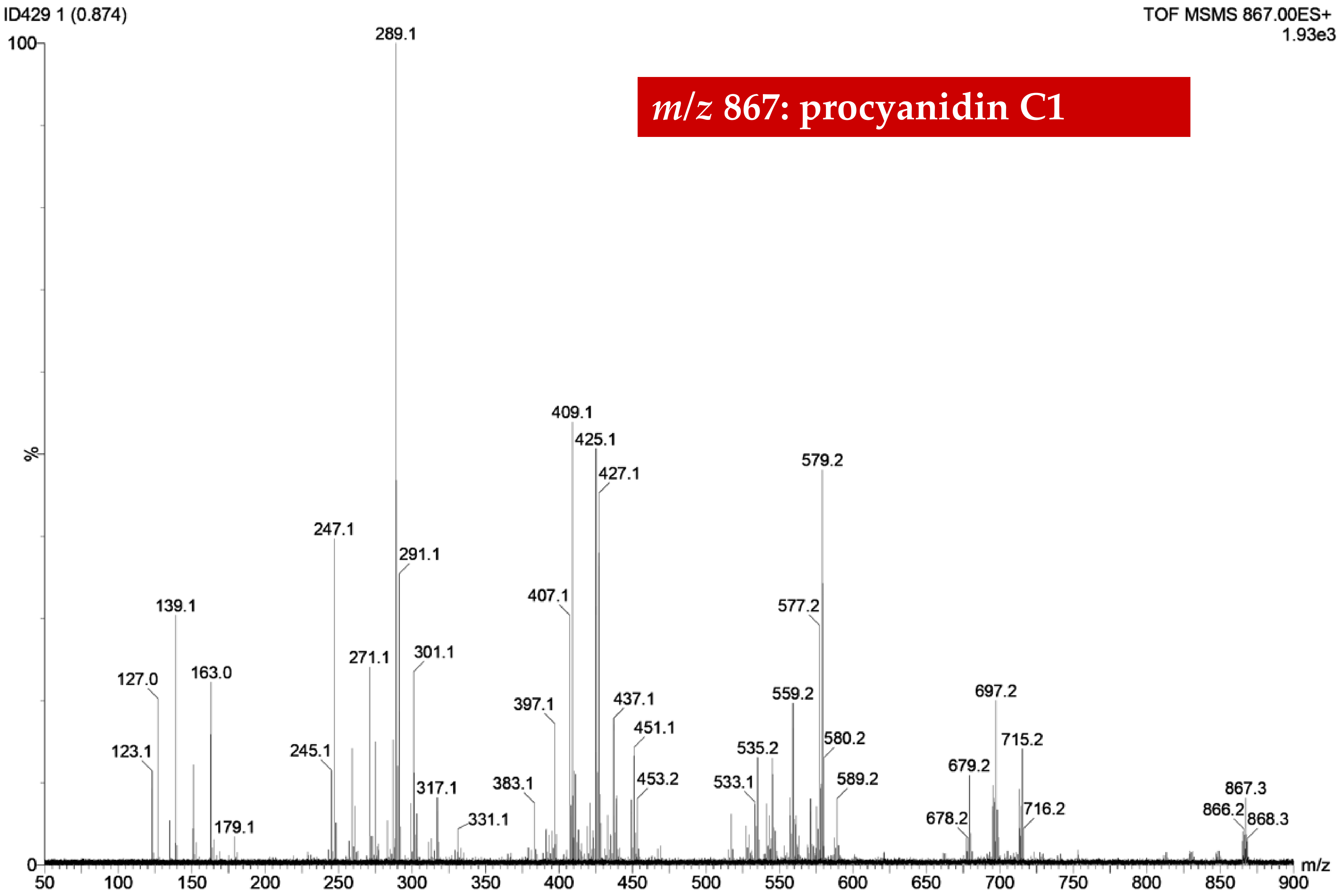

Supplementary Materials

Figure 7 - MS/MS spectrum at *m/z* 899 acquired in positive ion mode

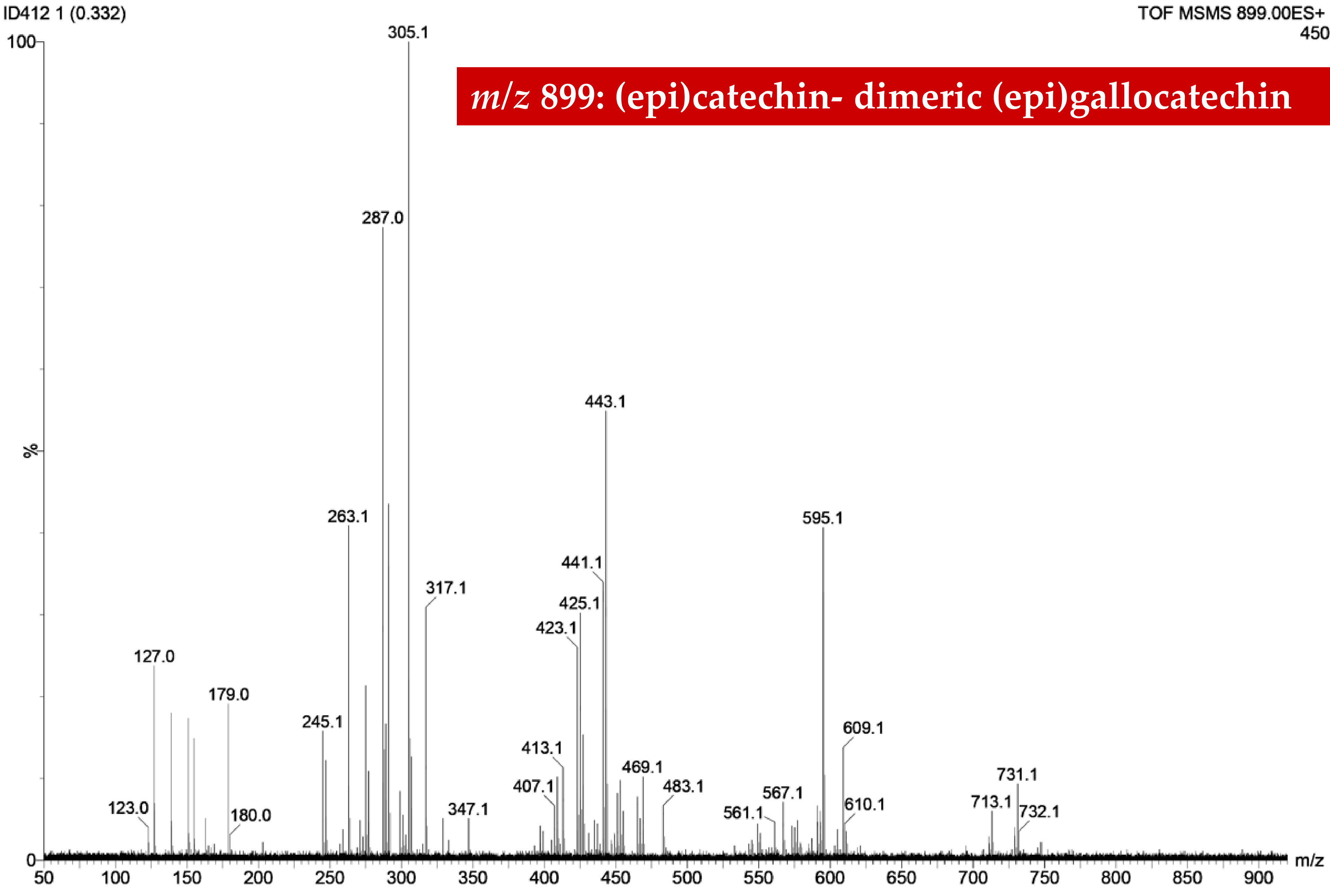

Supplementary Materials
Figure 8 - MS/MS spectrum at *m/z* 935 acquired in positive ion mode

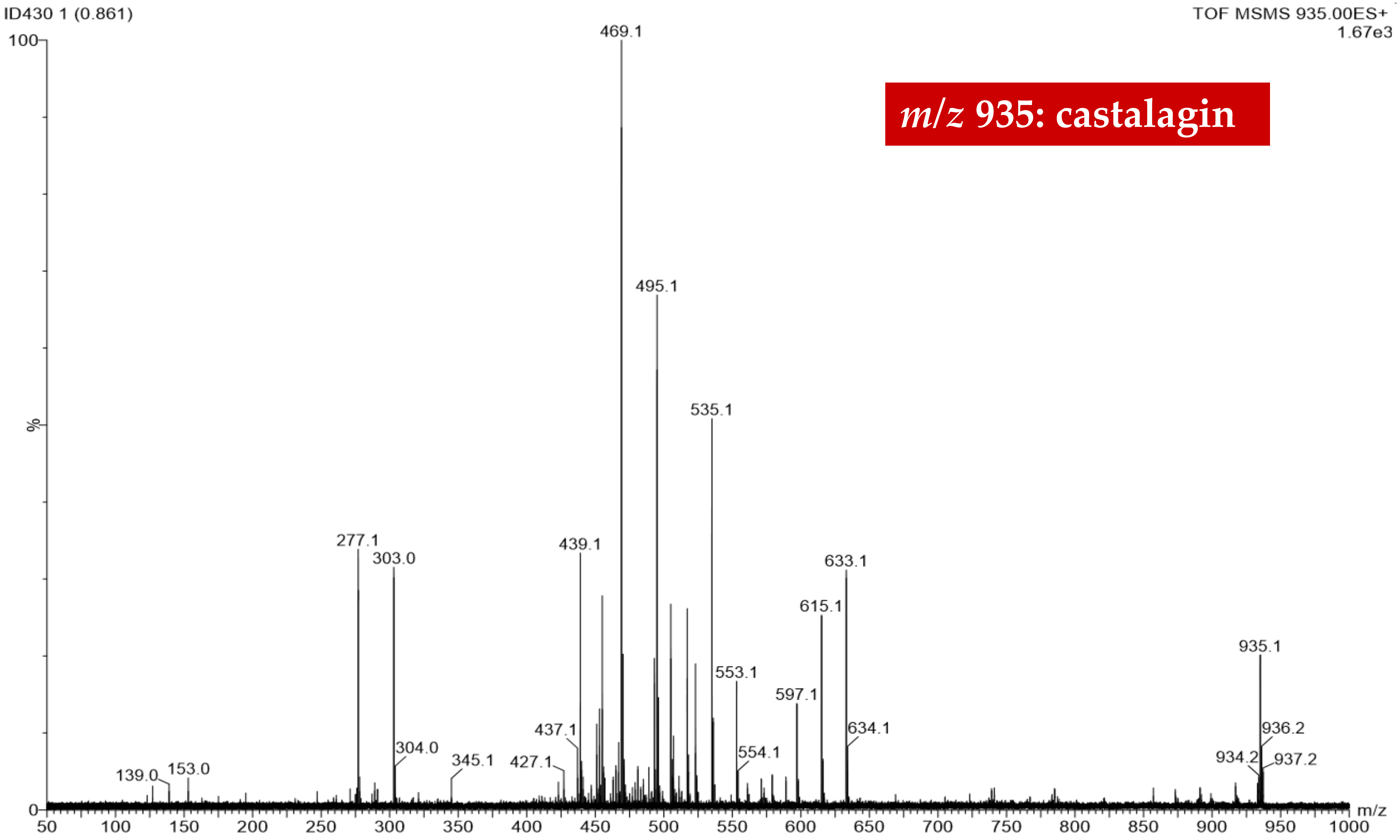

Supplementary Materials

Figure 9 - MS/MS spectrum at *m/z* 1019 acquired in positive ione mode

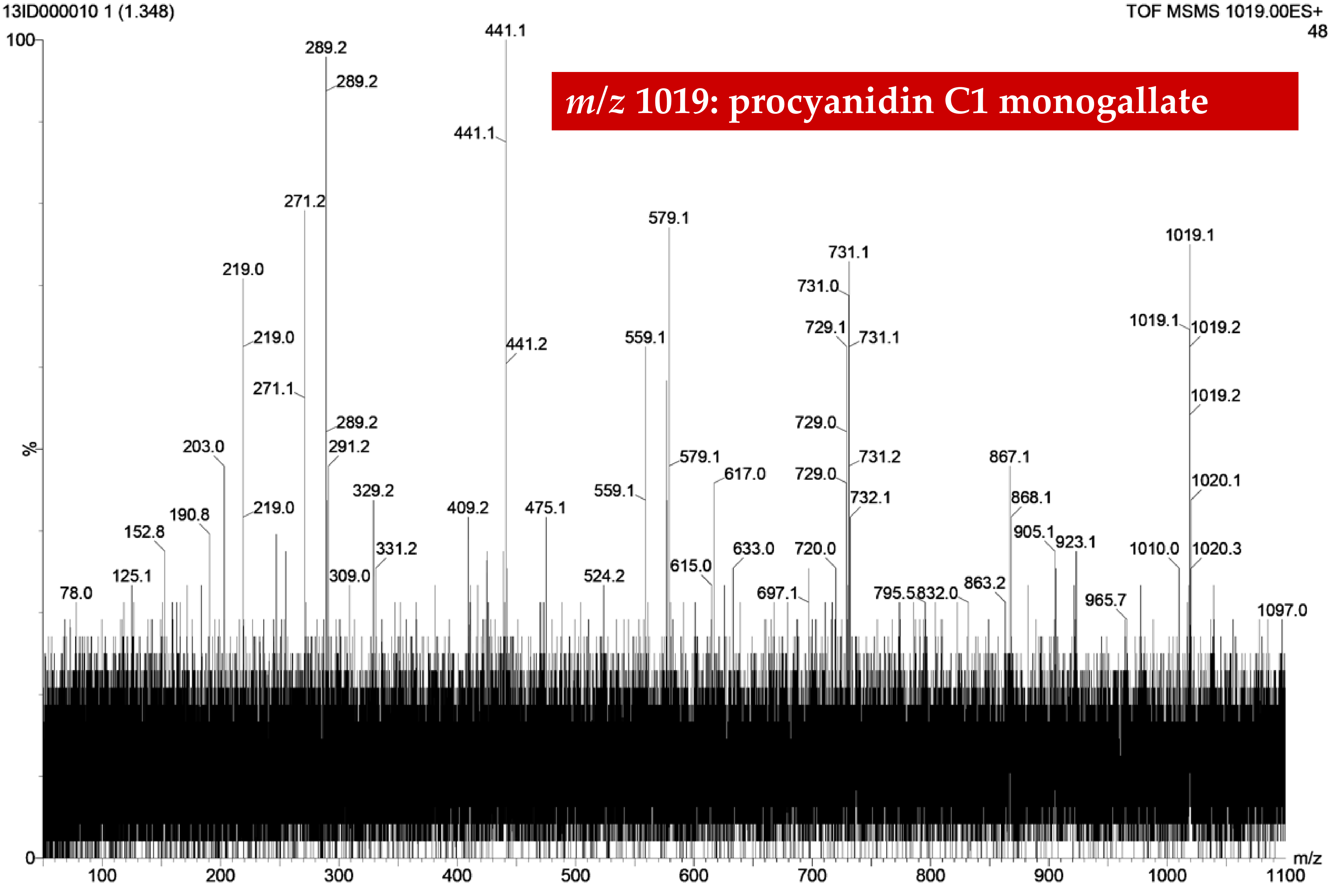

Supplementary Materials

Figure 10 - MS/MS spectrum at *m/z* 1155 acquired in positive ion mode

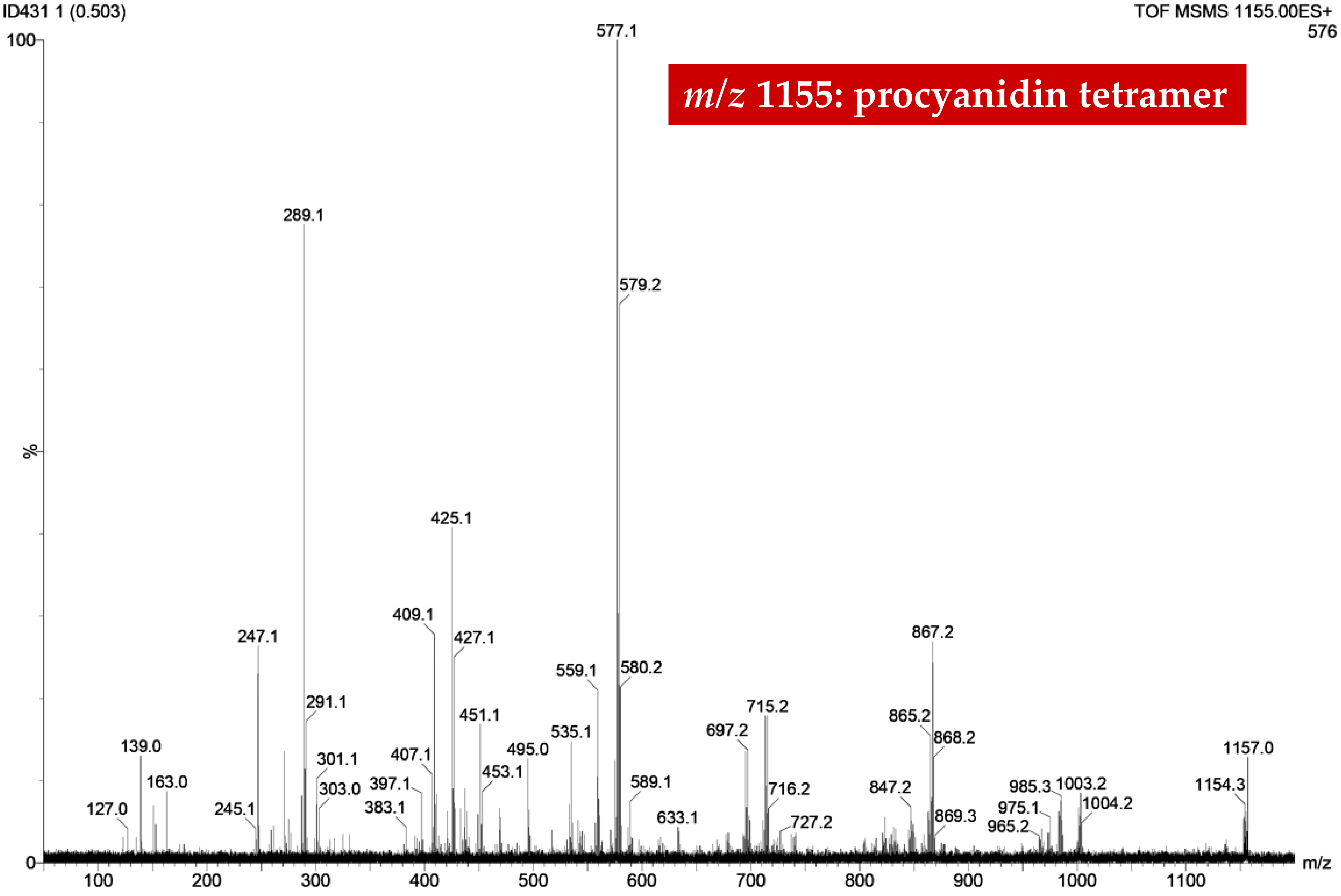

Supplementary Materials

Figure 11 - MS/MS spectrum at *m/z* 1203 acquired in positive ion mode

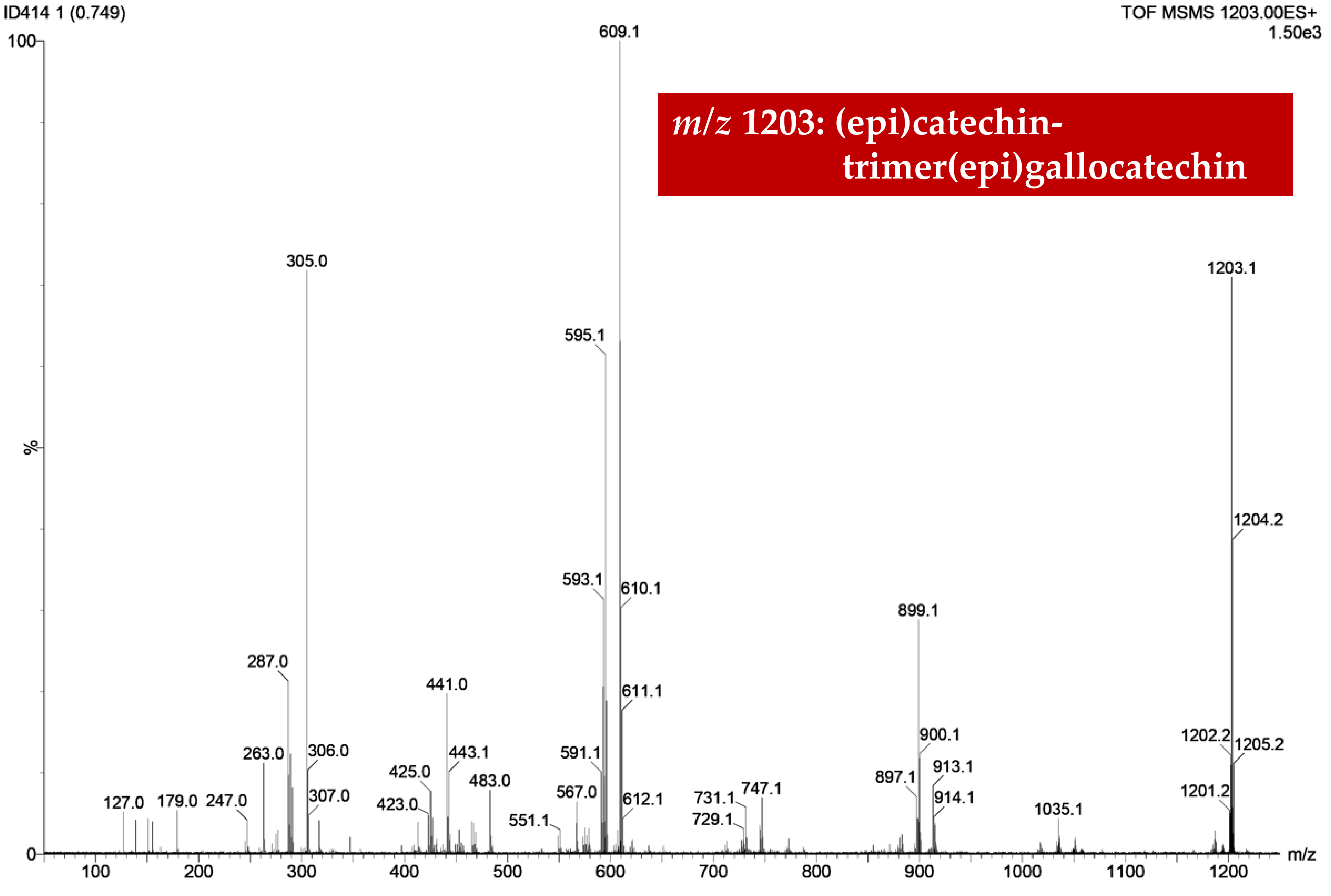

Supplementary Materials

Figure 12 - MS/MS spectrum at *m/z* 1307 acquired in positive ion mode

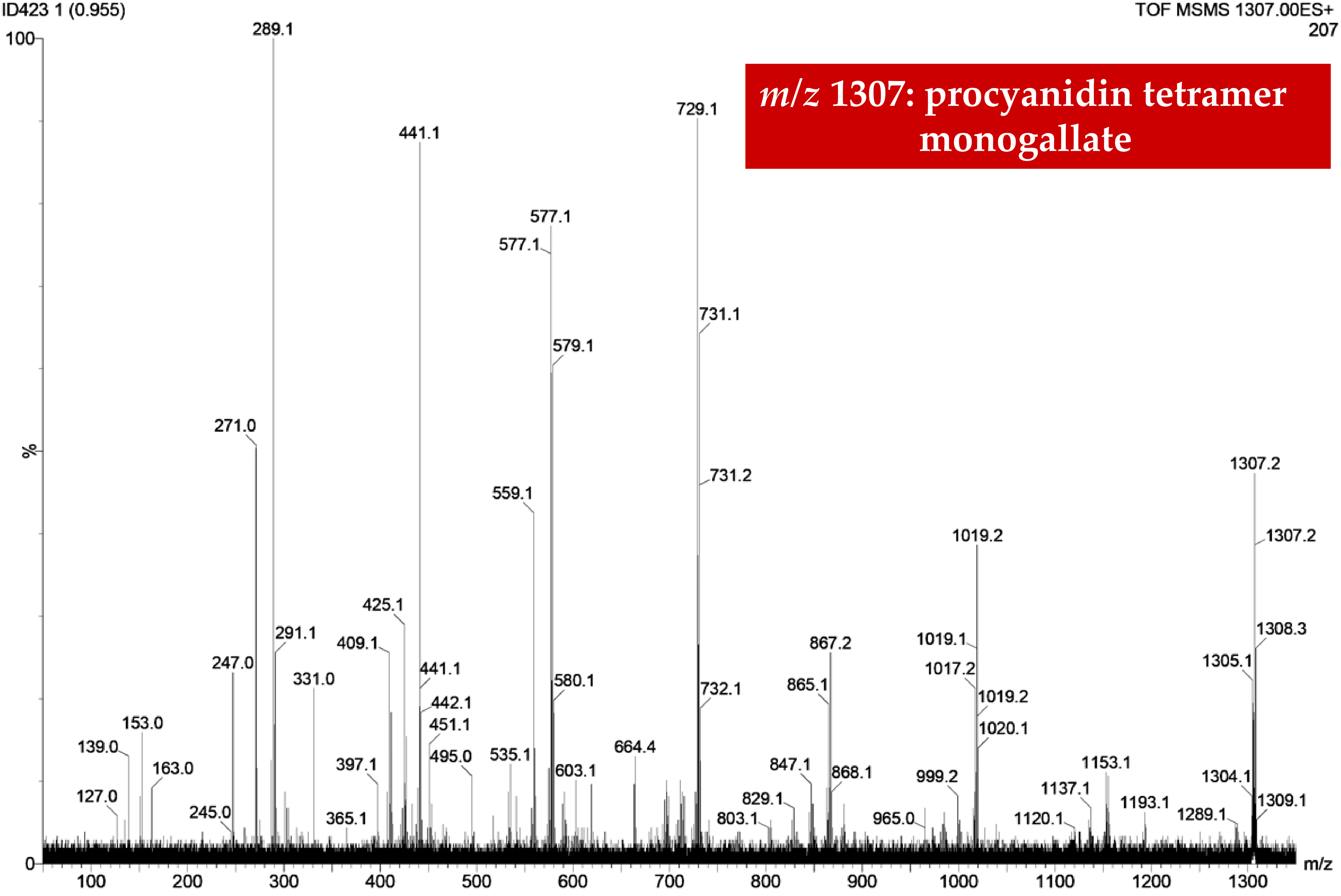

Supplementary Materials

Figure 13 - MS/MS spectrum at *m/z* 1443 acquired in positive ion mode

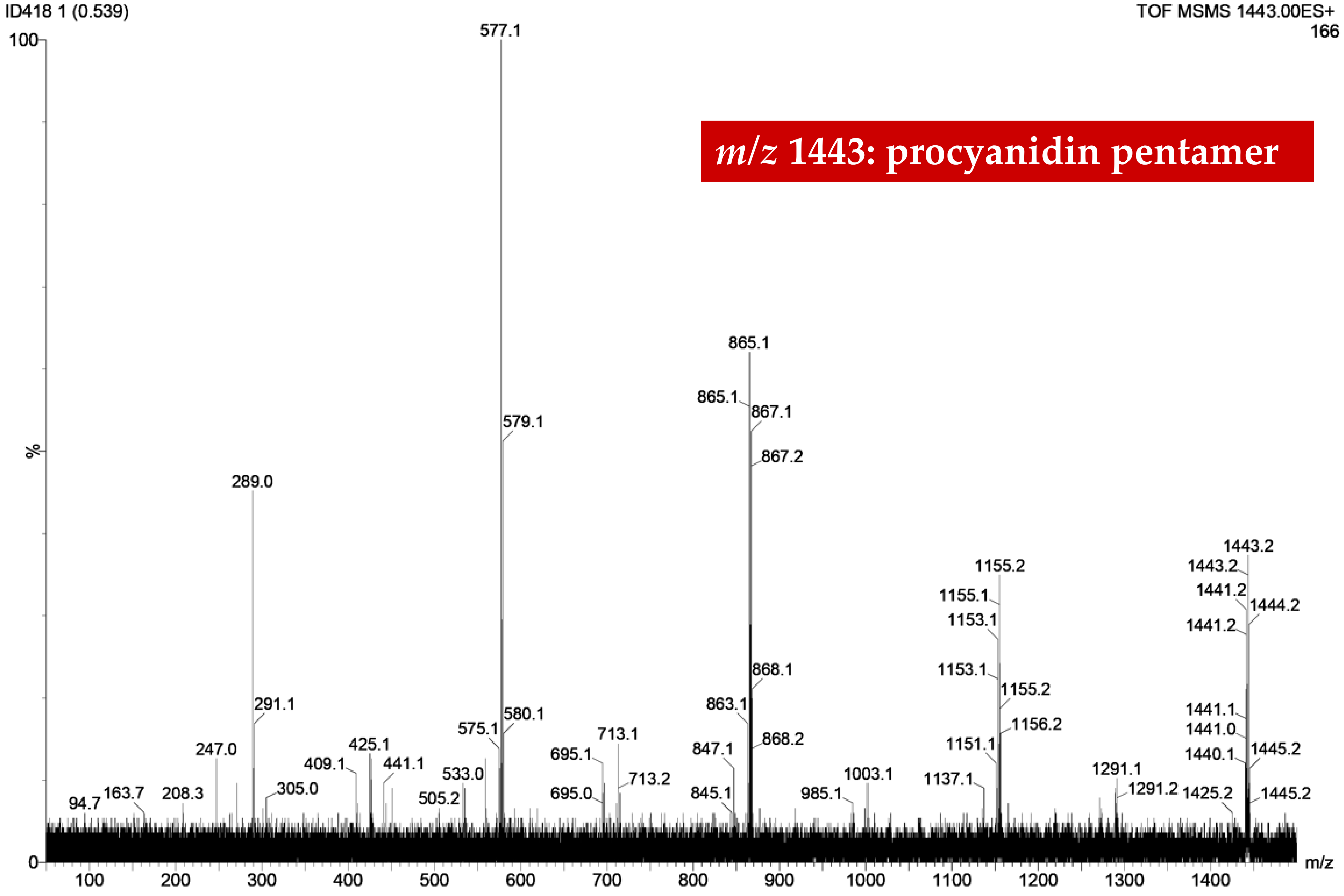

Supplementary Materials
Figure 14 - MS/MS spectrum at *m/z* 1507 acquired in positive ion mode

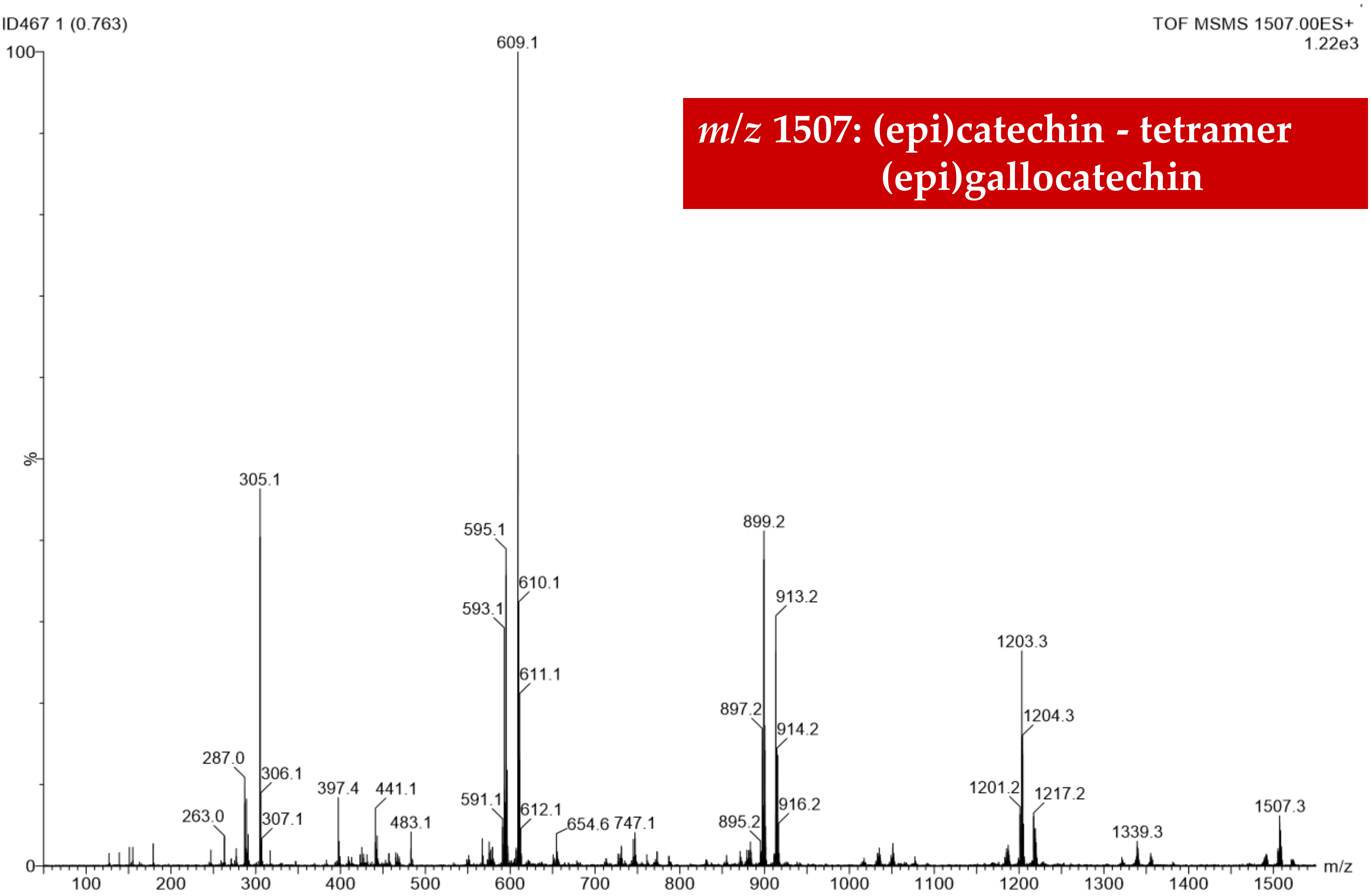

Supplementary Materials

Figure 15 - MS/MS spectrum at *m/z* 1595 acquired in positive ion mode

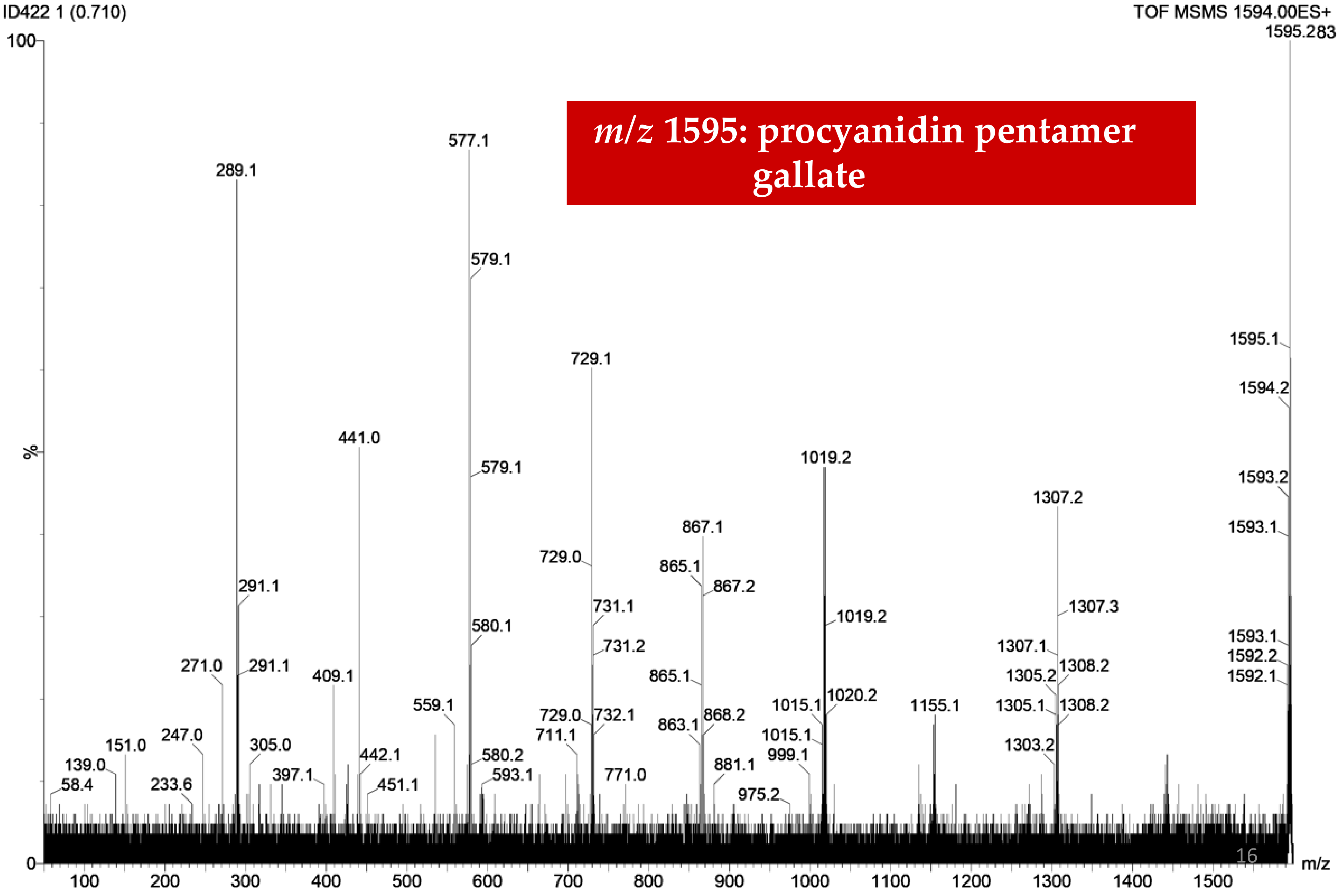

Supplementary Materials
Figure 16 - MS/MS spectrum at *m/z* 1747 acquired in positive ion mode

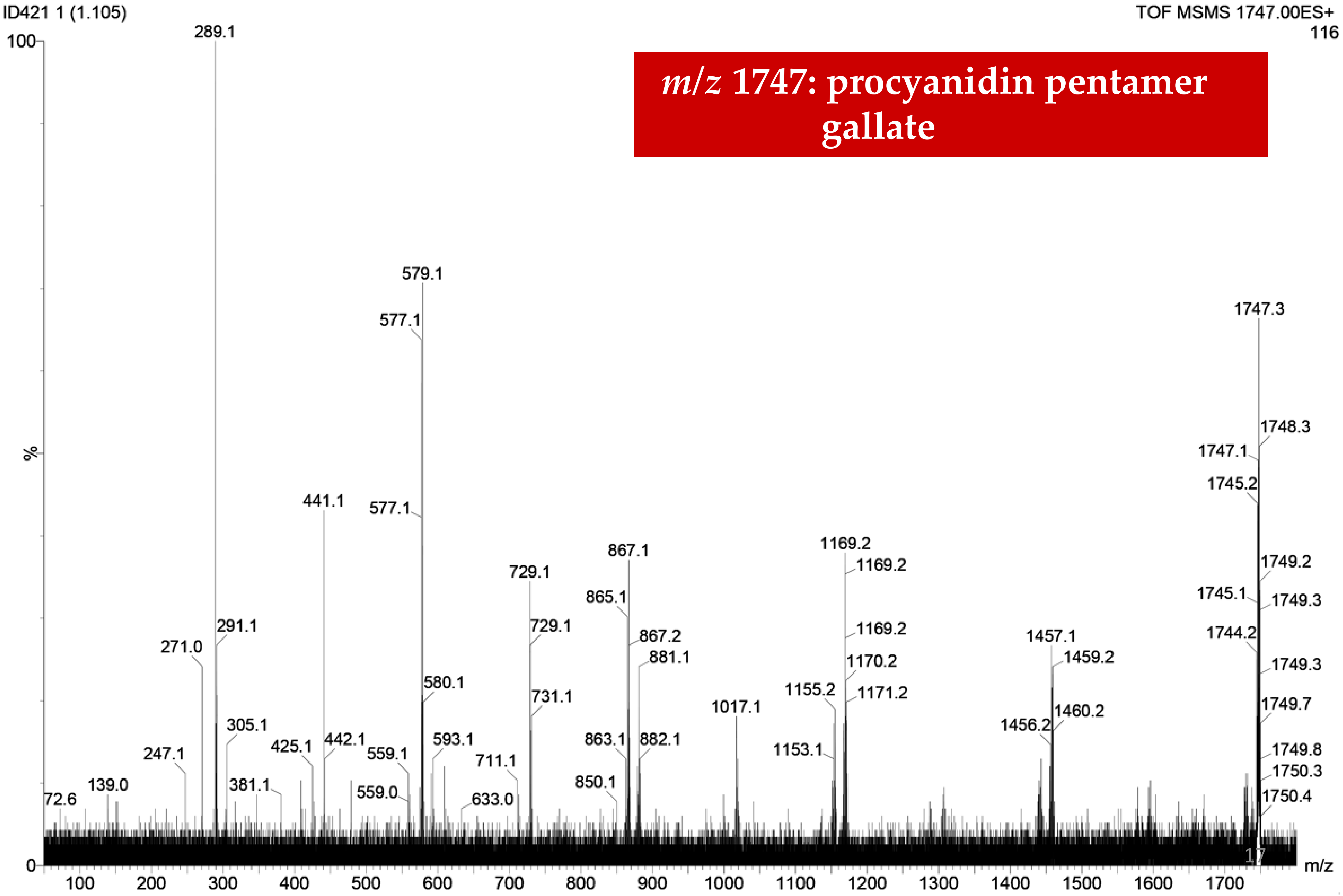

Supplementary Materials

Figure 17 - MS/MS spectrum at *m/z* 1811 acquired in positive mode

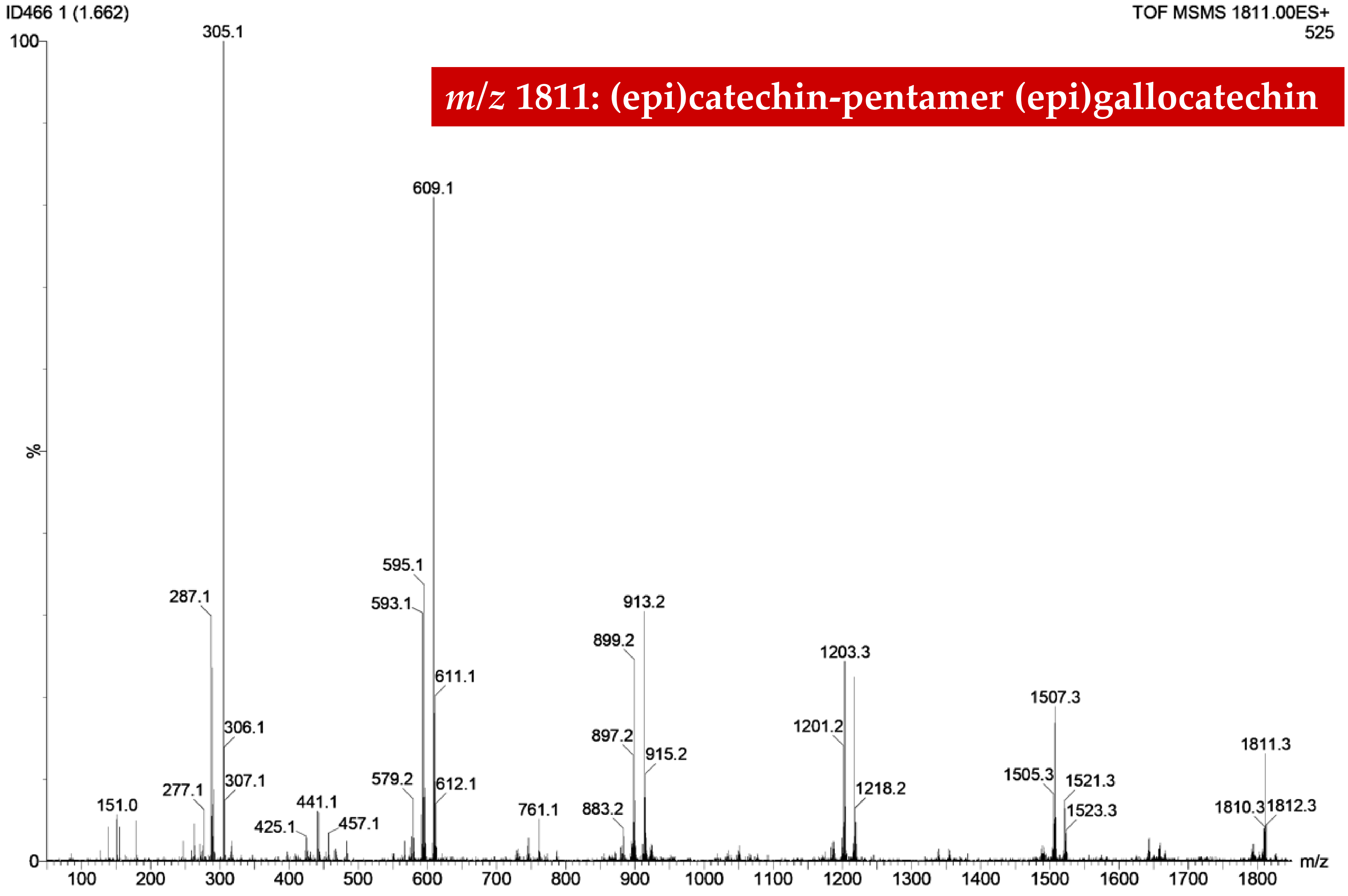

Supplementary Materials

Figure 18 - MALDI-TOF-MS in linear positive ion mode of wastewater samples from treatment with: A) tap water containing *L. pentosus*; B) tap water; C) recycled wastewater from a previous curing treatment with tap water.

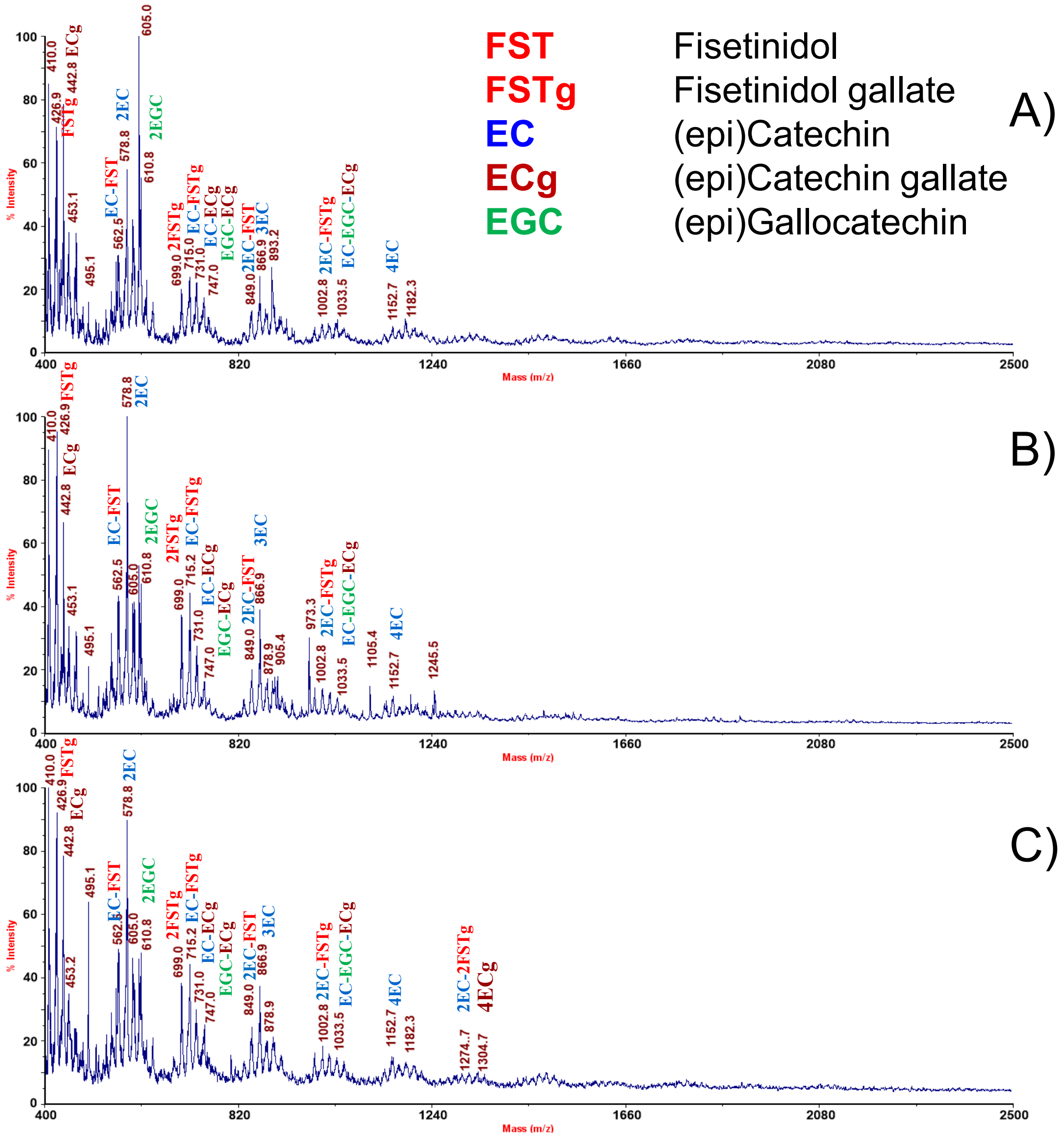

Supplement: Supplementary file 1 [file molecules-26-02335-s001.zip › molecules-1175216-supplementary.pdf]
